# Effectiveness and safety of consecutive single embryo transfer compared to double embryo transfer: results from the UK HFEA registry

**DOI:** 10.1093/humrep/deaf028

**Published:** 2025-02-25

**Authors:** Jack Tighe, Sophie Broughton, Rachel Roberts, Lorraine S Kasaven, Rachel Cutting, Elliot Bridges, Abigail Ng, Amanda Evans, Efstathios Theodorou, Jara Ben Nagi, Benjamin P Jones

**Affiliations:** Department of Gynaecology, Hammersmith Hospital, Imperial College Healthcare NHS Trust, London, UK; Department of Metabolism, Digestion and Reproduction, Institute of Reproductive & Developmental Biology, Imperial College London, Hammersmith Hospital Campus, London, UK; Department of Metabolism, Digestion and Reproduction, Institute of Reproductive & Developmental Biology, Imperial College London, Hammersmith Hospital Campus, London, UK; School of Medicine, University of Birmingham, Birmingham, UK; Department of Gynaecology, Hammersmith Hospital, Imperial College Healthcare NHS Trust, London, UK; Department of Gynaecology, Hammersmith Hospital, Imperial College Healthcare NHS Trust, London, UK; Centre for Reproductive and Genetic Health, London, UK; Human Fertility and Embryology Authority (HFEA), London, UK; Human Fertility and Embryology Authority (HFEA), London, UK; Human Fertility and Embryology Authority (HFEA), London, UK; Human Fertility and Embryology Authority (HFEA), London, UK; Centre for Reproductive and Genetic Health, London, UK; Centre for Reproductive and Genetic Health, London, UK; Department of Gynaecology, Hammersmith Hospital, Imperial College Healthcare NHS Trust, London, UK; Department of Metabolism, Digestion and Reproduction, Institute of Reproductive & Developmental Biology, Imperial College London, Hammersmith Hospital Campus, London, UK; The Lister Fertility Clinic, The Lister Hospital, London, UK

**Keywords:** embryo transfer, live birth, multiple birth, fertilization *in vitro*, pregnancy, multiple, birth rate, preterm birth, single embryo transfer, intracytoplasmic sperm injection

## Abstract

**STUDY QUESTION:**

How does two-consecutive single embryo transfer (2xSET) affect reproductive outcomes of IVF and ICSI compared to double embryo transfer (DET)?

**SUMMARY ANSWER:**

Two-consecutive SET may provide greater or comparable live birth rate (LBR); with lower multiple birth, preterm birth, and pregnancy loss or neonatal death rates compared to DET.

**WHAT IS KNOWN ALREADY:**

Elective SET in IVF/ICSI is widely encouraged over DET to minimize the risk of multiple births and associated morbidities. Despite this, multiple birth rates following IVF remain higher than the 10% target across Europe and the USA. Currently, the majority of evidence regarding SET and DET is based on various studies assessing outcomes such as LBR per treatment cycle, as opposed to per oocyte retrieval. As such, the representation of SET is mostly unfavourable. Analysis of cumulative LBR following the transfer of two embryos over consecutive cycles, rather than in one transfer event (DET) is more effective at distinguishing the two methods and will therefore provide more valuable information relevant to clinical practice.

**STUDY DESIGN, SIZE, DURATION:**

This retrospective cohort study was conducted using Human Fertilisation and Embryology Authority (HFEA) register data, which encompasses national data from all IVF clinics in the UK. All women who underwent their first oocyte retrieval and IVF or ICSI treatment cycle with subsequent SET, DET, or 2xSET between 2010 and 2019 using blastocyst embryos were included (N = 71 807).

**PARTICIPANTS/MATERIALS, SETTING, METHODS:**

The rate of live birth, liveborn baby rate, multiple birth, preterm birth, and pregnancy loss or neonatal death was compared between SET, DET, and 2xSET IVF/ICSI pregnancies using blastocyst-stage embryos, where data were stratified by maternal age. Data analysis was conducted in RStudio v4.2, alpha equals 0.05.

**MAIN RESULTS AND THE ROLE OF CHANCE:**

Blastocyst-stage 2xSET achieved a greater median LBR of 0.47 (interquartile range [IQR] 0.13) than SET, 0.41 (IQR 0.13), and DET, 0.38 (IQR 0.13) (*P* < 0.05). Using SET as the reference standard, 2xSET was associated with a significantly lower odds of multiple births compared to DET ((odds ratio [OR] 6.87, 95% CI 6.14–7.68) vs 28.20, 95% CI 25.20–31.57). The odds of preterm birth were also lower following 2xSET (OR 1.11, 95% CI 1.06–1.15) compared to DET (OR 2.80, 95% CI 2.67–2.94). Similarly, the odds of pregnancy loss or neonatal death were lower following 2xSET (OR 1.14, 95% CI 1.08–1.21) compared to DET (OR 2.11, 95% CI 1.98–2.24). LBR was consistently higher following 2xSET than DET and SET in women aged 39 years and under (*P* < 0.05). However, results were comparable in women over 39 years (*P* > 0.05). Across all age groups, DET pregnancies had the highest multiple birth rate (*P* < 0.05). In women aged 39 years and under, DET was associated with the highest preterm birth rate (*P* < 0.05), whereas the rate was comparable across cohorts in women over 39 (*P* > 0.05). Moreover, pregnancy loss and neonatal death rates were highest following DET in women aged 37 years and under (*P* < 0.05), and comparable to SET and 2xSET in women over 37 years (*P* > 0.05).

**LIMITATIONS, REASONS FOR CAUTION:**

Certain confounders are not recorded within HFEA registry data, including patient BMI, evaluation of embryo quality, and endometrial thickness at embryo transfer. Consequently, while our analysis identifies broad trends in embryo transfer success and morbidity, results may differ within certain patient populations.

**WIDER IMPLICATIONS OF THE FINDINGS:**

Blastocyst-stage 2xSET may provide greater LBR in women aged 39 years and under, and comparable LBR in women over 39 years old, with overall lower multiple birth and morbidity than DET. 2xSET should be considered first-line among certain patient cohorts, including women with advanced maternal age to improve reproductive outcomes and reduce the risk of morbidity following ART.

**STUDY FUNDING/COMPETING INTEREST(S):**

No external funding was used for this study. None of the authors has any conflicts of interest.

**TRIAL REGISTRATION NUMBER:**

This cohort study did not require registration. Following consultation with the Institutional Review Board at Imperial College London, ethical approval was not deemed necessary.

## Introduction

IVF is an increasingly common method used to achieve parenthood in subfertile couples, with over 76 000 IVF cycles undertaken in the UK in 2021 compared to just under 62 000 in 2011 (HFEA, 2013, 2023; Smeenk *et al.*, 2023). Since its implementation in the 1970s, there have been many advancements in ART techniques, including the introduction of ICSI in the mid-1990s, with an associated tripling of success rates over the last three decades (Niederberger *et al.*, 2018; HFEA, 2023). However, since its inception, multiple births remain the single biggest health risk from IVF treatment, with twin pregnancies accounting for 28% (1 in 4) of all IVF pregnancies in the 1990s (HFEA, 2023). There is extensive evidence that demonstrates multiple births are associated with increased adverse maternal and neonatal outcomes, increased financial burden, and a higher incidence of psychosocial issues such as maternal stress and depression compared to singleton births, as well as being an additional burden on the NHS (Jacklin and Marceniuk, 2018; ESHRE Guideline Group on the Number of Embryos to Transfer *et al.*, 2024). Promoting strategies to reduce the multiple birth rate, while maintaining or improving the live birth rate (LBR), is a primary objective of the Human Fertilisation and Embryology Authority (HFEA). Consequently, the number of multiple births has decreased to an average 5% (1 in 20) in 2021 (HFEA, 2023).

Elective single embryo transfer (SET) during IVF/ICSI, as opposed to the transfer of multiple embryos, is the most significant way to reduce the number of multiple births (Kamath *et al.*, 2020). Furthermore, the development of vitrification as a cryopreservation technique has greatly improved blastocyst survival and reproductive outcomes, with data demonstrating there is now no difference in ongoing pregnancy rates or LBRs between fresh and frozen embryo transfers (Zaat *et al.*, 2021). This has facilitated the opportunity to undertake cumulative SETs as a strategy to optimize success rates, while minimizing the risk of multiple gestation (Peng *et al.*, 2022). Importantly, there is now comparable cumulative LBR for SET compared to double embryo transfer (DET) (Peng *et al.*, 2022).

However, despite the overall reduction in transfer of multiple embryos, which has subsequently reduced multiple pregnancy rates across Europe and the USA, many countries continue to report multiple birth rates above the 10% target, as recommended by both the European Society of Reproduction and Embryology (ESHRE), and the HFEA (HFEA, 2022; ESHRE Guideline Group on the Number of Embryos to Transfer *et al.*, 2024; Smeenk *et al.*, 2023). Optimizing LBR, defined as the proportion of IVF cycles that result in a live birth, remains a challenge following elective SET. One of the limitations of current evidence is the discrepancy surrounding the interpretation of LBRs, where LBR per cycle is often referred to rather than cumulative LBR per oocyte retrieval which may result in an unfavourable representation of SET. Consequently, analysis of cumulative LBR may be more effective; that is comparing DET with two-consecutive SET (2xSET) from a single oocyte retrieval procedure.

The HFEA records a UK-wide registry of all IVF/ICSI cycles and outcomes, allowing for a large-scale comparison of SET, DET, and 2xSET. This national retrospective cohort study aims to explore current trends in LBRs, multiple births, and associated morbidity following SET, DET, and 2xSET in women undergoing IVF or ICSI in the UK. We hypothesize that blastocyst-stage 2xSET can achieve a cumulative LBR comparable to DET, with a lower rate of multiple births and associated morbidity including preterm births, pregnancy loss, and neonatal death.

## Materials and methods

This retrospective cohort study was conducted using HFEA registered data, which encompasses national data from all IVF clinics in the UK. Within this analysis, the records of all women who underwent their first oocyte collection and IVF or ICSI treatment cycle with subsequent SET, DET, or 2xSET between 2010 and 2019 using blastocyst-stage embryos were extracted. Of note, only women who underwent treatment using autologous oocytes were included in this study, excluding any cases of donor oocytes. The extracted data underwent a validation process conducted by the HFEA, whereby individual clinics were required to verify the accuracy of the reported data.

The SET and DET cohorts comprised fresh embryo transfers only. The 2xSET cohort comprised fresh embryo transfer followed by a frozen embryo transfer. Within this cohort, not all patients underwent a second treatment cycle and their reasons for withdrawal were not recorded in the HFEA registry. To mitigate the impact of incomplete follow-up on our analysis, second cycles in patients who did not complete their second SET cycle are assumed to be unsuccessful. This approach provides a cautious estimation of live birth and morbidity rates in the 2xSET cohort, while ensuring the results reflect the most reliable interpretation of the data.

Given the well-recognized negative effect of maternal age on IVF outcomes, the results were stratified by maternal age at first oocyte retrieval to enable meaningful interpretation of the results (Yan *et al.*, 2012). Accordingly, sub-analysis of treatment outcomes by patient age at oocyte retrieval was performed using the following age groups: women aged under 35, 35–37, 38–39, and over 39 years. There were no restrictions on the interval between oocyte retrieval and embryo transfer, or on the time between first and second embryo transfers for women who underwent 2xSET.

When outcomes occurred in less than five women for a given year or age group, the UK HFEA excluded this data prior to analysis to ensure adherence to data anonymity standards.

### Outcomes

The primary outcomes investigated in this study are LBR, liveborn baby rate (LBBR), multiple birth rate (MBR), and morbidity.

LBR was defined as the number of births with at least one liveborn child per patient. LBBR was defined as the total number of liveborn children per patient. MBR was defined as the proportion of live births resulting in two or more liveborn children.

Morbidity outcomes were divided into two groups: (i) preterm birth and (ii) pregnancy loss or neonatal death. Preterm birth rate was defined as the proportion of live births delivered before 37 + 0 weeks gestation. Pregnancy loss or neonatal death included miscarriage (foetal death or delivery of a dead foetus before 24 completed weeks of pregnancy), stillbirth (foetal death or delivery of a dead foetus after 24 completed weeks of pregnancy), intrauterine embryo reductions, ectopic pregnancy, terminations of pregnancy, and neonatal death within 30 days of birth. Pregnancy loss or neonatal death rate was subsequently defined as the proportion of live births which resulted in pregnancy loss or neonatal death.

### Statistical analysis

RStudio v4.2 was used for data analysis. Sample distribution was evaluated using the Shapiro–Wilk test. Descriptive statistical analysis was described as median ± interquartile range (IQR). Mann–Whitney *U* test for unpaired non-parametric data was performed to determine statistical significance of both LBR and LBBR between groups. In instances where outcomes included both parametric and non-parametric cohort data, non-parametric statistical analysis and data presentation were undertaken to ensure rigorous statistical evaluation of significance and prevent comparison between median and mean values. Odds ratio (OR) and 95% CIs were calculated for multiple birth, preterm birth, and pregnancy loss or neonatal death rates, using unconditional maximal likelihood estimation, which aims to minimize bias in large non-parametric datasets (Milligan, 2003; Luque-Fernandez *et al.*, 2018).

Kruskal–Wallis test for non-parametric data was performed to determine statistical significance between cohorts when stratified by age, with further analysis between individual cohorts undertaken using the Mann–Whitney *U* test. In cases where data suppression resulted in less than three viable datasets within a cohort, or no instances of live birth, multiple birth, or morbidity were reported, the Mann–Whitney *U* test was unable to be performed and significance was identified using Kruskal–Wallis alone. *P*-values <0.05 were considered statistically significant.

### Ethical approval

Ethical approval was not required for this study following consultation with the institutional review board at Imperial College London.

## Results

Seventy-one thousand eight hundred and seven women from 105 IVF centres, who underwent embryo transfer with blastocyst-stage embryos during their first treatment cycle in the UK between 2010 and 2019 were included in this analysis (Table 1). One IVF centre was excluded owing to technical issues in data reporting.

**Table 1.** Demographic characteristics of patients.

	Blastocyst transfer procedure		
	Single (SET)	Double (DET)	2xSET
**Age** (mean ± SEM)	32.5 ± 0.018	35.6 ± 0.029	32.5 ± 0.018
**Ethnicity** (%)			
White	68.6	62.9	68.6
Asian	12.3	11.7	12.3
Black	2.3	3.8	2.3
Other	2.0	2.8	2.0
Mixed	1.4	1.6	1.4
Not Stated	13.4	17.1	13.4
**Gravidity** (mean ± SEM)			
Miscarriages	0.04127 ± 0.00093	0.07644 ± 0.00190	0.05621 ± 0.00111
TOP	0.00350 ± 0.00028	0.00404 ± 0.00047	0.00437 ± 0.00031
Other (including ectopic pregnancy)	0.00019 ± 0.00006	0.00051 ± 0.00015	0.00027 ± 0.00007
**Infertility cause** (%)			
Male factor	35.8	33.7	35.9
Female factor			
Ovulatory	14.0	10.5	14.0
Tubal	13.1	11.6	13.2
Uterine	0.9	1.3	0.9
Endometriosis	6.6	6.1	6.6
Unexplained	32.6	34.0	32.7
Combined	89.6	86.7	89.7
**ART method** (%)			
IVF	50.5	46.4	50.5
ICSI	49.5	53.6	49.5
**Number of oocytes retrieved**	13.08 ± 0.026	12.18 ± 0.036	13.08 ± 0.026
(Mean ± SEM)			

Patient characteristics for SET, DET, and 2xSET cohorts undergoing blastocyst-stage embryo transfers, SET N = 48 050, DET N = 23 757, 2xSET N = 48 049. Abbreviations: SET, single embryo transfer; DET, double embryo transfer; 2xSET, two-consecutive single embryo transfers; TOP, termination of pregnancy.

In the 2xSET group (N = 48 050) 98.29% of patients received two-consecutive blastocyst-stage embryo transfers. In the remaining 1.71% of women who did not have further treatment after one SET cycle, the analysis assumed no live birth resulted from the second cycle.

### Live births

**Figure 1.**
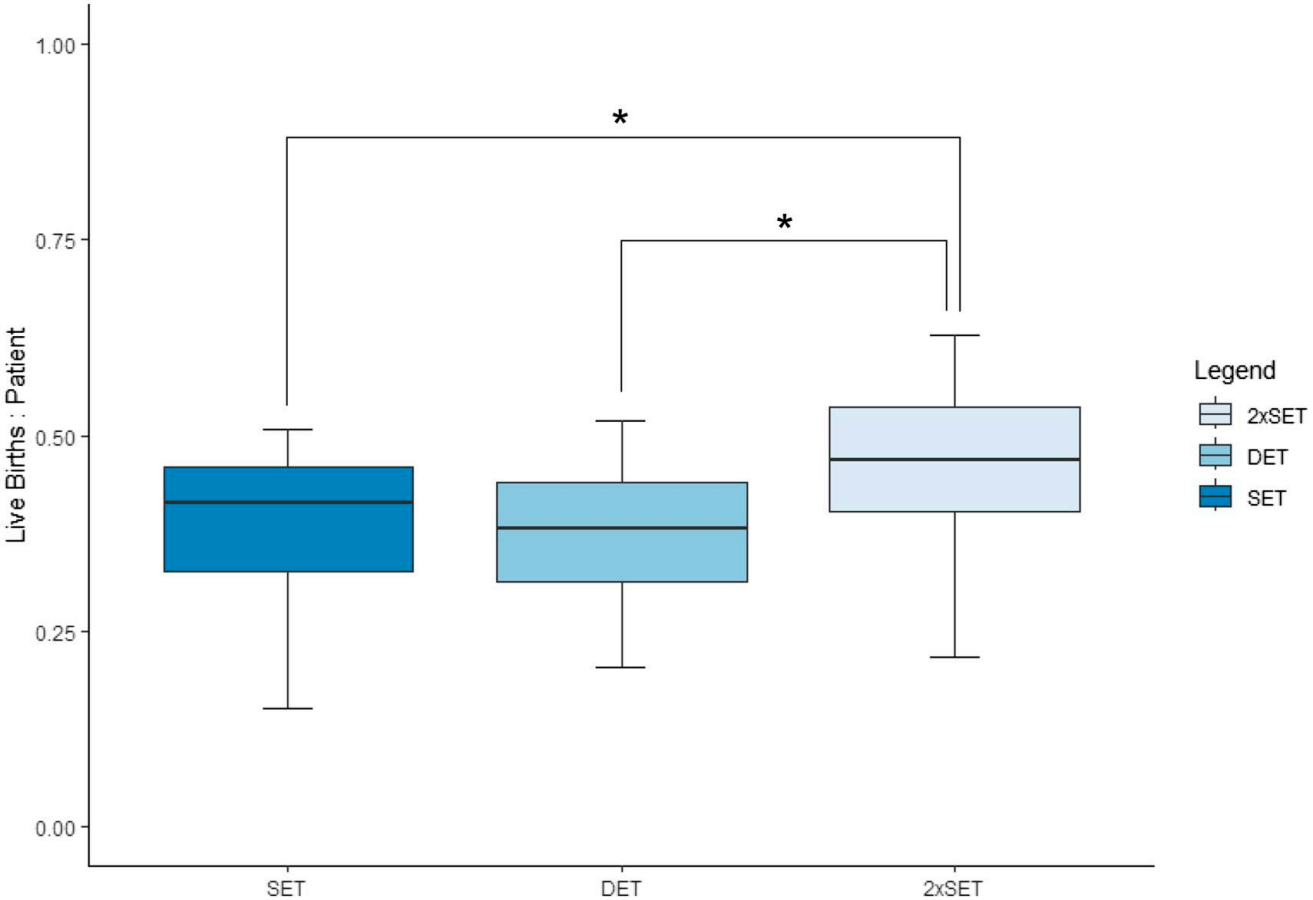
**Boxplot of live births per patient in women receiving blastocyst-stage embryo transfers.** Range is represented by the outer two lines in each plot, outliers are represented by black dots, interquartile range is represented by the outer edges of each box and median is represented by the horizontal line within each box plot. Mann–Whitney *U* test for unpaired non-parametric data performed with black line indicating significant difference between groups (*P < 0.05). SET N = 48 050, DET N = 23 757, 2xSET N = 48 049. SET, single embryo transfer; DET, double embryo transfer; 2xSET, two-consecutive single embryo transfer cycles.

In women receiving blastocyst-stage embryo transfer, LBR did not significantly differ between SET and DET, demonstrating rates of 0.41 (IQR 0.13) and 0.38 (IQR 0.13), respectively (*P* > 0.05) (Fig. 1). However, the 2xSET estimate was higher than either SET or DET, with a rate of 0.47 (IQR 0.13) (*P* < 0.05).

**Figure 2.**
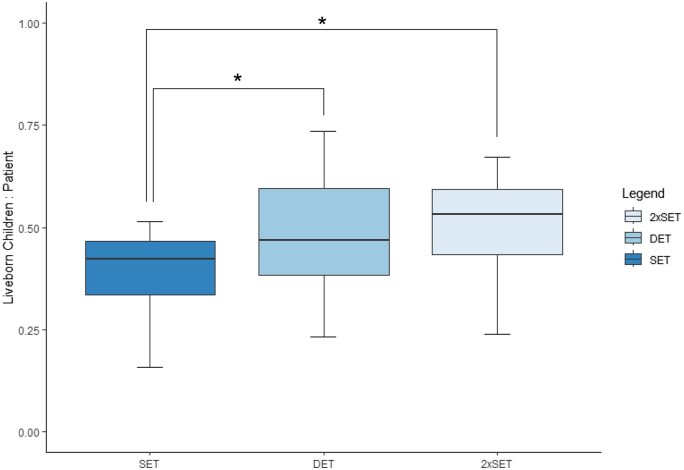
**Boxplot of liveborn children per patient in women receiving blastocyst-stage embryo transfers.** Range is represented by the outer two lines in each plot, outliers are represented by black dots, interquartile range is represented by the outer edges of each box and median is represented by the horizontal line within each box plot. Mann–Whitney *U* test for unpaired non-parametric data performed with black line indicating significant difference between groups (*P < 0.05). SET N = 48 050, DET N = 23 757, 2xSET N = 48 049. SET, single embryo transfer; DET, double embryo transfer; 2xSET, two-consecutive single embryo transfer cycles.

Blastocyst-stage SET demonstrated a significantly lower LBBR compared to DET, reporting rates of 0.42 (IQR 0.13) and 0.47 (IQR 0.21), respectively (*P* < 0.05) (Fig. 2). The 2xSET cohort demonstrated an LBBR greater than SET, achieving rates of 0.53 (IQR 0.16) (*P* < 0.05).

### Multiple births and morbidity

Compared to SET, the odds of multiple births, preterm births and pregnancy loss, or neonatal death among women receiving blastocyst-stage embryo transfers was higher in DET (Table 2). DET reported an OR of 28.20 (95% CI 25.20–31.57) for multiple birth, 2.80 (95% CI 2.67–2.94) for preterm birth, and 2.11 (95% CI 1.98–2.24) for pregnancy loss or neonatal death, compared to SET pregnancies.

**Table 2.** Odds ratios for multiple birth and pregnancy outcomes.

Stage	Cohort	**Multiple birth** OR (95% CI)	**Preterm birth** OR (95% CI)	**Pregnancy loss or neonatal death** OR (95% CI)
Blastocyst	SET	REF	REF	REF
	DET	28.20 (25.20–31.57)	2.80 (2.67–2.94)	2.11 (1.98–2.24)
	2xSET	6.87 (6.14–7.68)	1.11 (1.06–1.15)	1.14 (1.08–1.21)

Odds ratio for multiple birth, preterm birth, and pregnancy loss or neonatal death outcomes in SET, DET, and 2xSET cohorts in women receiving blastocyst-stage embryo transfers. Odds ratio and 95% CI has been calculated compared to a reference cohort (denoted by REF). SET N = 25 119, DET N = 10 718, 2xSET N = 30 246.

SET, single embryo transfer; DET, double embryo transfer; 2xSET, two-consecutive single embryo transfer cycles; OR, odds ratio; 95%, REF, reference cohort.

Blastocyst-stage 2xSET reported higher odds of multiple births when compared to SET, OR 6.87 (95% CI 6.14–7.68), however, this was much lower compared to DET, OR 28.20 (95% CI 25.20–31.57) (Table 2).

The odds of preterm birth were greater following blastocyst-stage 2xSET than SET, OR 1.11 (95% CI 1.06–1.15). Similarly, the odds of pregnancy loss or neonatal death were increased following 2xSET, OR 1.14 (95% CI 1.08–1.21) (Table 2).

### Maternal age

**Figure 3.**
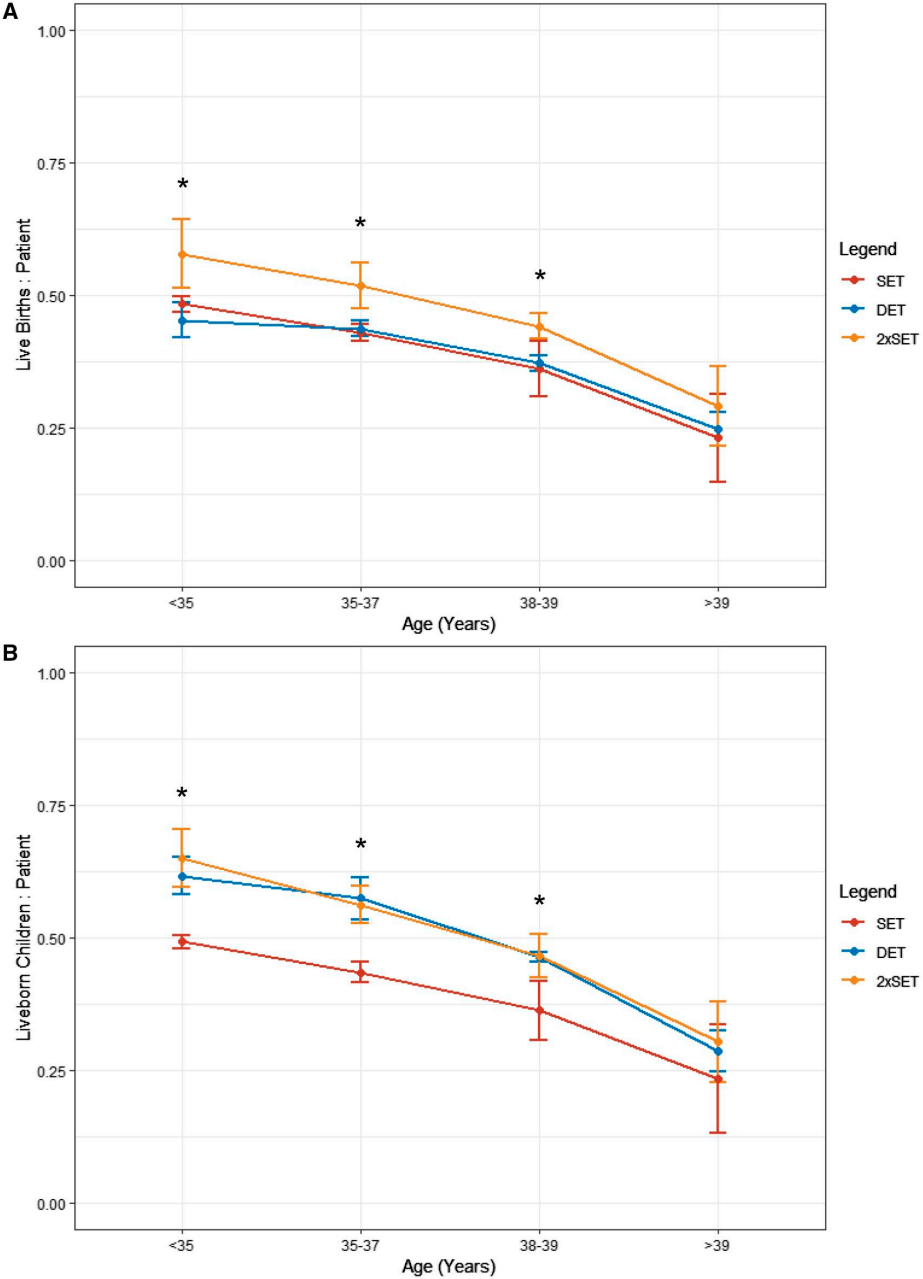
**Line plots of live birth rate, and liveborn baby rate in women receiving blastocyst-stage embryo transfers, stratified by age.** (**A**) live births per patient and (**B**) liveborn children per patient. Each cohort can be identified by the following colour: SET (red), DET (blue), and 2xSET (amber).Results are stratified into four age groups: under 35, 35–37, 38–39, and over 39 years. Points are median ± IQR. Kruskal–Wallis’s test was performed with significance between cohorts within each age group denoted with an Asterix (*P < 0.05). SET N = 48 050, DET N = 23 757, 2xSET N = 48 049. SET, single embryo transfer; DET, double embryo transfer; 2xSET, two-consecutive single embryo transfer cycles; IQR, interquartile range.

Blastocyst-stage 2xSET achieved the highest LBR in women aged 39 years and under (*P* < 0.05) (Fig. 3 and Supplementary Fig. S1). In patients over 39 years old, LBR was comparable between SET, DET, and 2xSET (*P* > 0.05) (Supplementary Fig. S1).

In patients aged 39 years and under, LBBR was higher in blastocyst-stage DET and 2xSET cohorts compared to SET (*P* < 0.05) (Fig. 3 and Supplementary Fig. S2). LBBR was comparable between SET, DET, and 2xSET in women over 39 years old (Supplementary Fig. S2).

**Figure 4.**
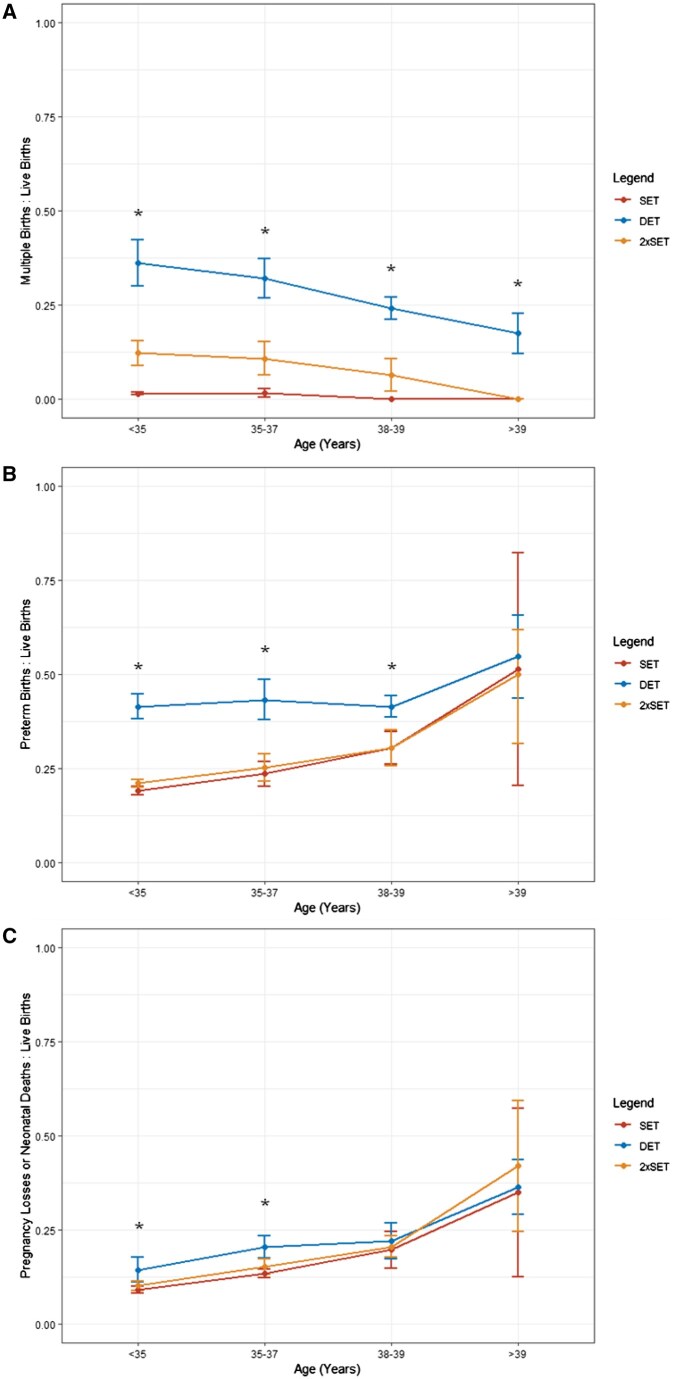
**Line plots of multiple birth rate, preterm birth rate, and pregnancy loss or neonatal death rate for women receiving blastocyst-stage embryo transfers, stratified by age.** (**A**) multiple births to live births, (**B**) preterm births to live births, and (**C**) pregnancy losses or neonatal deaths to live births. Each cohort can be identified by the following colour: SET (red), DET (blue), and 2xSET (amber). Results are stratified into four age groups: under 35, 35–37, 38–39, and over 39 years. Points are median ± IQR. Kruskal–Wallis’s test was performed with significance between cohorts within each age group denoted with an Asterix (*P < 0.05). SET N = 25 119, DET N = 10 718, 2xSET N = 30 246. SET, single embryo transfer; DET, double embryo transfer; 2xSET, two-consecutive single embryo transfer cycles; IQR, interquartile range.

Multiple birth, preterm birth, and pregnancy loss or neonatal death rates in women receiving blastocyst-stage embryo transfer varied significantly by age (Fig. 4). DET continuously had the highest rate of multiple births compared to SET and 2xSET across all four age groups (Supplementary Fig. S3). Additionally, DET reported the highest preterm birth rate in women aged 39 years and under compared to SET and 2xSET (Supplementary Fig. S4). Moreover, DET had the highest rate of pregnancy loss or neonatal death in women aged 37 years and under. However, no significant difference in pregnancy loss or neonatal death rate between cohorts was observed in women aged 39 years and over (Supplementary Fig. S5).

## Discussion

This is one of the largest studies (N = 71 807) to report that 2xSET is safer, and specifically in women 39 years and under, more effective than DET, thereby providing greater insights into the success and morbidity rates following IVF or ICSI treatment in the UK. Compared to DET, 2xSET, at the blastocyst stage, resulted in a higher LBR in women 39 years and under, and comparable LBR in women over 39 years, with lower multiple birth and morbidity rates. These results align with the findings of Mejia *et al.* (2021) and are consistent with the UK HFEA and National Institute for Health and Care Excellence (NICE) guidance, as well as recent ESHRE clinical guidelines promoting elective SET (NICE, 2013; HFEA, 2022; ESHRE Guideline Group on the Number of Embryos to Transfer *et al.*, 2024).

The success of consecutive SET has previously been observed in numerous randomized controlled trials, with a meta-analysis by Kamath *et al.* (2020) reporting similar cumulative LBRs between DET and consecutive cleavage-stage embryo transfers (N = 985, four studies). Our study findings in blastocyst-stage embryos are consistent with this wider literature. However, we also demonstrate that, depending on maternal age, 2xSET may result in comparable, or even superior, LBR compared to DET while also reducing the rate of multiple births and associated morbidity (Figs 3 and 4). These findings support the recent ESHRE 2023 guidance advocating for consecutive SET irrespective of embryo quality. Conversely, the NICE guidance suggests DET may be appropriate in women aged 37–39 years old, following the assessment of embryo quality (NICE, 2013; ESHRE Guideline Group on the Number of Embryos to Transfer *et al.*, 2024). Unfortunately, embryo quality is not recorded within the HFEA registry, therefore we could not stratify outcomes by this parameter. In a recent retrospective cohort study by Chen *et al.* (2020), LBR was comparable in women 35 and over receiving SET or DET with average-quality frozen-thawed blastocysts, suggesting transfer of multiple embryos does not improve outcomes in embryos of average quality. However, in those women aged over 35 years old, the average age was 37 years (Chen *et al.*, 2020). As such, the results may be less applicable to women of more advanced age (Chen *et al.*, 2020). As our analysis did not report outcomes based on embryo quality using standardized measures such as the Gardener Criteria, our findings cannot be specifically extrapolated to women with either good or average embryo quality (Gardner *et al.*, 2000; Chen *et al.*, 2020; Lai *et al.*, 2020). Further studies evaluating the effect of embryo quality on 2xSET outcomes, particularly in women of advanced maternal age, could provide valuable insights to guide clinical practice. Nonetheless, given the increased risk of multiple births, associated morbidity, and subsequent financial burden associated with DET, 2xSET offers a promising alternative to DET that may achieve comparable success rates in women over 39 years old, while mitigating these risks (NICE, 2013; HFEA, 2022; ESHRE Guideline Group on the Number of Embryos to Transfer *et al.*, 2024).

DET was associated with higher risk of preterm birth and neonatal death or pregnancy loss compared to SET and 2xSET pregnancies, likely due to the higher rate of multiple births (Fig. 4). Especially as twin pregnancies are associated with poorer maternal and neonatal outcomes compared to singleton (Eapen *et al.*, 2020). A recent multinational systematic review analysed maternal outcomes for IVF or ICSI twins versus singletons and demonstrated that the incidence of antenatal hospitalization and postpartum haemorrhage in twin pregnancies was doubled compared to singleton (Eapen *et al.*, 2020). Similarly for neonatal outcomes, the rates of low birth weight infants were increased 10-fold and admission to neonatal intensive care was over six times higher among twins compared to singletons (Eapen *et al.*, 2020). Additionally, an increasing number of studies are evaluating the long-term outcomes of multiple births on child development and health. Thus far, evidence suggests that multiple births arising from IVF are at increased risk of neurodevelopmental disabilities, particularly cerebral palsy, as well as cardiovascular disorders and certain cancers (Chen and Heilbronn, 2017; Kuiper *et al.*, 2017; Briana and Malamitsi-Puchner, 2019).

Our study identified that although the odds of preterm birth were lower in blastocyst-stage 2xSET compared to DET, preterm birth was more likely following 2xSET compared to SET alone (Table 2). Preterm birth can be caused by various factors and is generally classified as either spontaneous or iatrogenic (Cavoretto *et al.*, 2018). While these two classifications are generally reported as one outcome, as is the case within this HFEA registry data, the risk factors, management, and prognosis can vary significantly between subtypes (Jackson *et al.*, 2004; McDonald *et al.*, 2005; Pandey *et al.*, 2012; Qin *et al.*, 2016; Cavoretto *et al.*, 2018). Consequently, although the causes of preterm birth were not identified within this analysis, the findings may be attributed to several reasons, including differences in the time between embryo transfers, changes in patient characteristics between transfers, including BMI, impaired glucose regulation, and hypertensive disease, as well as changes to the indications for medical premature delivery (Torloni *et al.*, 2009; McDonald *et al.*, 2010; Cavoretto *et al.*, 2018; Berger *et al.*, 2020; Tang *et al.*, 2020). Therefore, although 2xSET is less likely to result in preterm birth than DET, further research is required to identify those who are at increased risk during the second SET transfer.

Advancing maternal age is independently associated with obstetric complications such as gestational diabetes, hypertensive disorders of pregnancy, placenta praevia, and maternal thrombosis (Timofeev *et al.*, 2013). Consequently, while 2xSET may offer a safer alternative to DET, individual patient risk at the time of transfer must be carefully assessed to avoid complications. This is particularly important given the potential for a prolonged interval between the first and second embryo transfer in 2xSET, during which a woman’s risk profile may change significantly due to ageing (Timofeev *et al.*, 2013). Moreover, prolonged embryo cryopreservation has been associated with reduced LBR, further highlighting the importance of timely embryo transfer to minimize the risk of complications while maximizing the chance of live birth (Zhang *et al.*, 2021). Unfortunately, age at embryo transfer, and time interval between the first and second embryo transfer were not available for analysis within this present dataset. Further evaluation of patient outcomes stratified by such variables could offer valuable insights and establish clearer guidelines on appropriate age limits for embryo transfer.

Our findings clearly demonstrate that 2xSET provides a comparable LBR in women over 39 years, and more successful LBR in women 39 years and under, with concurrent lower multiple birth rate and reduced morbidity than DET. However, a 2021 nationwide HFEA patient survey (N = 1233) reported that 30% of responding patients in the UK undergo multiple embryo transfer during IVF/ICSI treatment (HFEA, 2021). ESHRE 2023 guidelines suggest couples should receive comprehensive fertility counselling prior to treatment to understand the risks of multiple births and that 2xSET reduces multiple birth occurrence compared to DET (ESHRE Guideline Group on the Number of Embryos to Transfer *et al.*, 2024). However, qualitative evidence has highlighted the lack of patient understanding regarding the risks of multiple births. Many couples prefer twins over singletons unless the success rates of SET are presented as comparable to those of multiple embryo transfer (Leese and Denton, 2010). A systematic review and meta-analysis by Sunderam *et al.* (2018) has demonstrated how comprehensive patient education can improve patient preference for SET and plays a significant role in reducing DET uptake. Our data demonstrate that 2xSET at blastocyst stage has not just comparable, but higher LBR than DET in women aged 39 years and under, which could be important to highlight when counselling patients.

Economic analysis has shown that SET is more cost-effective than DET, given the increased antenatal healthcare costs associated with multiple births (Scotland *et al.*, 2011). Although DET may initially appear less expensive than 2xSET, by reducing the number of treatment cycles, the long-term financial burden outweighs the upfront savings due to the significantly increased pregnancy/infant-related medical expenses associated with multiple births due to increased morbidity (Crawford *et al.*, 2016). Ensuring equal access to IVF or ICSI treatment is crucial for the effective implementation of SET policies. Limited access to fertility treatment, whether due to financial or cultural factors, can influence patients to choose multiple embryo transfer rather than SET (Adamson and Norman, 2020). Additionally, patients frequently underestimate the costs and psychosocial impacts of multiple births (Howard, 2018). Parents of twins under 5 years often experience poorer parent–child interactions compared to those with singletons (Anderson *et al.*, 2016). Additionally, mothers of twins are at higher risk of experiencing maternal stress and depression (Ellison *et al.*, 2005; Baor and Soskolne, 2012).

Although this study reports various demographic characteristics of the patients, including ethnicity, previous miscarriages, infertility aetiology, and the number of oocytes retrieved (Table 1), subgroup analysis was limited to patient age as this data were available from the HFEA registry. Moreover, certain confounders were not recorded in the HFEA registry, including patient BMI, embryo quality, endometrial thickness at embryo transfer, and embryo transfer protocol. Additionally, while the HFEA registry records whether treatments were state-funded or privately funded, this specific variable was not available for sub-analysis in the current dataset. Furthermore, clinical practice may vary between clinics, and the decision to undergo DET could have been influenced by several factors including previous miscarriages, embryo quality, and patient choice. Moreover, as the reasons for not continuing with a second SET cycle are not recorded within the HFEA registry, our results may represent a conservative estimate of 2xSET success. Consequently, while our analysis identifies broad trends in embryo transfer success and morbidity, results may differ within certain patient populations, therefore prospective studies are required to evaluate SET, DET, and 2xSET outcomes within select patient populations (Bellver *et al.*, 2010; Lv *et al.*, 2020; Barad *et al.*, 2022).

Embryo quality is likely a key factor influencing the decision between SET and DET, with variations in clinical practice across fertility clinics. Patients with lower-quality embryos may be considered unsuitable for cryopreservation, depending on clinic protocols, and are therefore excluded from undergoing 2xSET. This could introduce a selection bias, as patients undergoing 2xSET may predominantly have higher-quality embryos, resulting in higher success rates (Lv *et al.*, 2020; Tomic *et al.*, 2020). However, the comparable LBBR between the 2xSET and DET cohorts in women aged 38 years and over, suggest that if DET patients underwent 2xSET instead, they would experience similar LBRs and morbidity outcomes, providing embryo attrition associated with cryopreservation and thawing does not preclude a second embryo transfer cycle (Fig. 2) (Coello *et al.*, 2021). To validate this hypothesis, further assessment of the outcomes following 2xSET is required, implementing a standardized approach, where embryo quality can be stratified.

We acknowledge that the data were limited with respect to analysing the order of multiple births, such as twins versus triplets, higher order pregnancies, and for combining occurrence of pregnancy loss or neonatal death due to low sample sizes. In addition, while HFEA registry data endeavours to be as complete and accurate as possible, omission of data required to preserve patient anonymity for this study has led to limited analysis within certain areas, particularly regarding multiple birth and morbidity rates. Future analysis of such data could be undertaken through an application to the HFEA register research panel, where omitted data could be analysed. Furthermore, despite attempts to include all neonatal deaths following IVF or ICSI treatment, it remains potentially underreported within the UK HFEA registry data. Moreover, while this remains a very large UK national dataset (N = 71 807), 2019 live birth data are currently classified as preliminary data within the HFEA registry at the time of this analysis. Consequently, future studies utilizing HFEA registry data from 2019 may report slight variations in results.

## Conclusion

In women over 39 years old 2xSET may provide a similar LBR to DET and in women aged 39 years and under, higher cumulative LBRs with lower multiple birth and morbidity rates than DET. Consequently, a preference for 2xSET at blastocyst-stage embryo transfer could improve LBR while reducing multiple gestations and improving maternal and neonatal outcomes. Clinical guidance should therefore encourage SET to emphasize the benefits of two-consecutive SETs with advancing maternal age. Further research regarding the potential impact of embryo quality on 2xSET outcomes is recommended to enable individualized clinical management and to make national and international recommendations and good practice points regarding embryo transfer.

## Supplementary Material

deaf028_Supplementary_Figure_S1

deaf028_Supplementary_Figure_S2

deaf028_Supplementary_Figure_S3

deaf028_Supplementary_Figure_S4

deaf028_Supplementary_Figure_S5

## Data Availability

The data underlying this article were provided by the UK Human Fertilisation and Embryology Authority (HFEA) under licence/by permission. Data will be shared on request to the corresponding author with permission of the UK HFEA.
