# Supplementary material for: Effectiveness and safety of consecutive single embryo transfer compared to double embryo transfer: results from the UK HFEA registry
Source: Hum Reprod. 2025 Feb 25;40(5):885–94. doi: 10.1093/humrep/deaf028 (PMC12046072; doi:10.1093/humrep/deaf028)
Supplement: deaf028_Supplementary_Figure_S1 [file deaf028_supplementary_figure_s1.pdf]

OXFORD
human reproduction
Human Reproduction, 2025, 1–1
https://doi.org/10.1093/humrep/deaf028
Supplementary Data

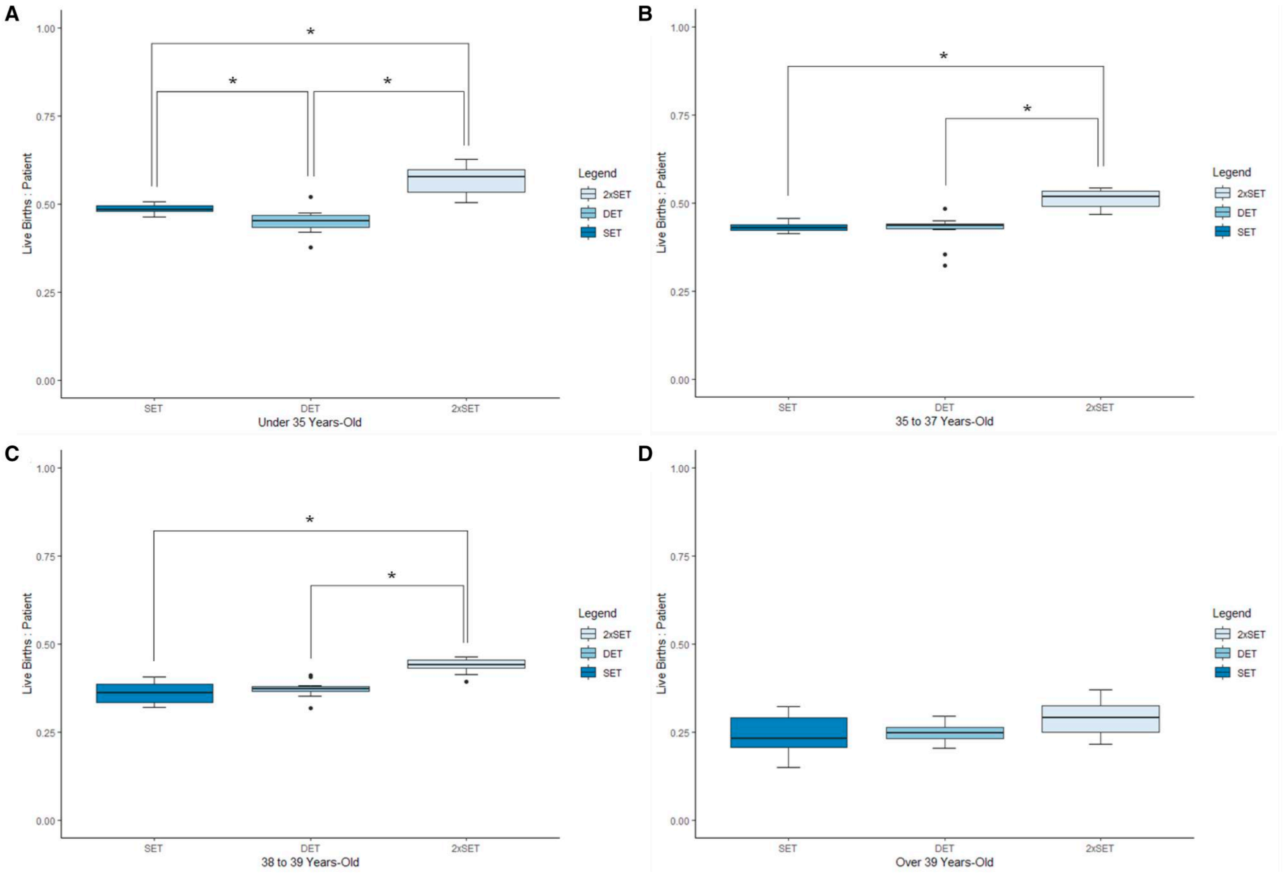

Supplementary Figure S1. Boxplots of live births per patient in women receiving blastocyst-stage embryo transfers stratified by age. (**A**) Under 35 years old, (**B**) 35–37 years old, (**C**) 38–39 years old, and (**D**) over 39 years old. Range is represented by the outer two lines in each plot, outliers are represented by black dots, interquartile range is represented by the outer edges of each box and median is represented by the horizontal line within each box plot. Mann–Whitney *U* test for unpaired non-parametric data performed with black line indicating significant difference between groups (*$P < 0.05$). Black line indicating significant difference between groups (*$P < 0.05$). SET N = 48 050, DET N = 23 757, 2xSET N = 48 049. SET, single embryo transfer; DET, double embryo transfer; 2xSET, two-consecutive single embryo transfer cycles.
